# Trends in Nanotechnology and Its Potentialities to Control Plant Pathogenic Fungi: A Review

**DOI:** 10.3390/biology10090881

**Published:** 2021-09-08

**Authors:** Abdulaziz Bashir Kutawa, Khairulmazmi Ahmad, Asgar Ali, Mohd Zobir Hussein, Mohd Aswad Abdul Wahab, Abdullahi Adamu, Abubakar A. Ismaila, Mahesh Tiran Gunasena, Muhammad Ziaur Rahman, Md Imam Hossain

**Affiliations:** 1 Department of Plant Protection, Faculty of Agriculture, Universiti Putra Malaysia, Serdang 43400, Malaysia; mohdaswad@upm.edu.my (M.A.A.W.); gs52595@student.upm.edu.my (A.A.); gs54934@student.upm.edu.my (A.A.I.); gs57269@student.upm.edu.my (M.T.G.); gs53254@student.upm.edu.my (M.Z.R.); gs53259@student.upm.edu.my (M.I.H.); 2 Department of Biological Sciences, Faculty of Life Science, Federal University Dutsin-Ma, Dutsin-ma P.M.B 5001, Nigeria; 3 Sustainable Agronomy and Crop Protection, Institute of Plantation Studies (IKP), Universiti Putra Malaysia, Serdang 43400, Malaysia; 4 Centre of Excellence for Postharvest Biotechnology (CEPB), School of Biosciences, University of Nottingham Malaysia, Jalan Broga, Semenyih 43500, Malaysia; 5 Institute of Advanced Technology (ITMA), Universiti Putra Malaysia, Serdang 43400, Malaysia; mzobir@upm.edu.my; 6 Department of Biological Sciences, Faculty of Science, Sokoto State University, Birnin Kebbi Road, Sokoto P.M.B 2134, Nigeria; 7 Department of Integrated Science, School of Secondary Education (Science), Federal College of Education (Technical), Bichi P.M.B 3473, Nigeria; 8 Grain Legume and Oil Crop Research and Development Centre, Angunakolapelessa 82220, Sri Lanka; 9 Plant Pathology Division, Regional Agricultural Research Station (RARS), Bangladesh Agricultural Research Institute (BARI), Barishal 8211, Bangladesh

**Keywords:** biosafety, disease control, essential oils, fungi, nanocarrier, nanoparticle, nanopesticides, nanotechnology

## Abstract

**Simple Summary:**

Fungal pathogens were reported to cause about 70–80% losses in yield. Nanotechnology can be a panacea to this problem by reducing the negative effect of the fungicides, enhancing the solubility of low water-soluble fungicides, and reducing their toxic effect in a sustainable and eco-friendly manner. This review focuses on the description, properties, and synthesis of nanoparticles, their utilization for plant pathogenic fungal disease control (either in the form of nanoparticles alone, or in the form of a nanocarrier for several fungicides), nano-formulations of agro-nanofungicides, *Zataria multiflora*, and ginger essential oils to control plant pathogenic fungi, as well as the biosafety and limitations of the nanoparticles applications.

**Abstract:**

Approximately 15–18% of crops losses occur as a result of animal pests, while weeds and microbial diseases cause 34 and 16% losses, respectively. Fungal pathogens cause about 70–80% losses in yield. The present strategies for plant disease control depend transcendently on agrochemicals that cause negative effects on the environment and humans. Nanotechnology can help by reducing the negative impact of the fungicides, such as enhancing the solubility of low water-soluble fungicides, increasing the shelf-life, and reducing toxicity, in a sustainable and eco-friendly manner. Despite many advantages of the utilization of nanoparticles, very few nanoparticle-based products have so far been produced in commercial quantities for agricultural purposes. The shortage of commercial uses may be associated with many factors, for example, a lack of pest crop host systems usage and the insufficient number of field trials. In some areas, nanotechnology has been advanced, and the best way to be in touch with the advances in nanotechnology in agriculture is to understand the major aspect of the research and to address the scientific gaps in order to facilitate the development which can provide a rationale of different nanoproducts in commercial quantity. In this review, we, therefore, described the properties and synthesis of nanoparticles, their utilization for plant pathogenic fungal disease control (either in the form of (a) nanoparticles alone, that act as a protectant or (b) in the form of a nanocarrier for different fungicides), nano-formulations of agro-nanofungicides, *Zataria multiflora*, and ginger essential oils to control plant pathogenic fungi, as well as the biosafety and limitations of the nanoparticles applications.

## 1. Introduction

Agriculture plays a vital role by providing nourishment and serving as a source of income for many countries. It is the major source of livelihood for people in rural areas; about 86% of the rural people depend on agricultural cultivation [1]. Approximately 15–18% of crops losses occur as a result of animal pests, while weeds and microbial diseases cause 34 and 16% losses, respectively. Fungal pathogens cause about 70–80% losses in yield [1]. Approximately, there are 1.5 million species that are classified under the kingdom ‘fungi’ and these fungal pathogens are mostly parasitic and saprophytic in nature, causing different diseases in agricultural crops. Fungal pathogens may cause serious decreases in the yield of different crops worldwide each year [2,3]. Presently, disease control depends on the utilization of agrochemicals, for example, fungicides. Regardless of numerous favorable advantages, such as fast action, reliability, and high availability, fungicides can cause negative impacts on the non-target living organisms because of their toxicity and their systemic mode of action by disrupting the metabolite levels in the biosynthetic pathway of aromatic amino acids within the soil microorganisms, the development of resistance, and resurgence in the population of pests and environment [4,5]. Moreover, it is assessed and estimated that about 80–90% of sprayed fungicides are lost to the environment after or during their applications [5,6]. Accordingly, there is an urgent need to procure high-performance fungicides which are cost-effective and cause less negative impacts to the environment.

**Figure 1:**
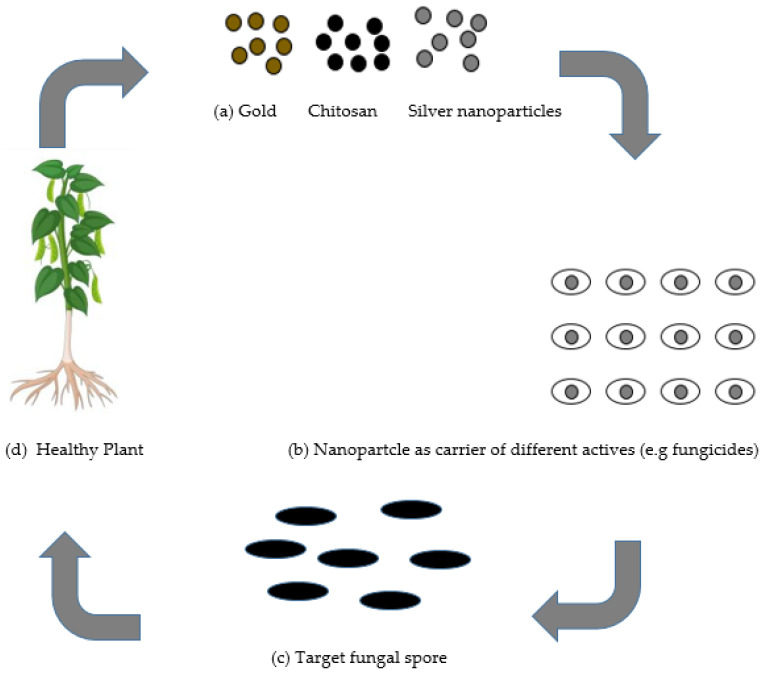
(**a**) Different nanomaterials as protectants used in plant protection. (**b**) Nanomaterials as transporters for several active ingredients such as fungicides. (**c**) Nanomaterials targeting different fungal pathogens. (**d**) The potentialities of nanomaterials to provide protection to the plant.

Nanotechnology has prompted the advancement of novel ideas and agriproducts having tremendous potential to address the aforementioned issues. Nanotechnology has progressed in areas of pharmacology and medicine, yet has not developed nearly as much in agricultural uses [7]. The utilization of nanotechnology in the agricultural sector is presently being investigated in the delivery of plant chemical, water, and seed control, nanobarcoding, transfer of genes, controlled release of agrochemicals, and nanosensors [8]. Many researchers have designed nanoparticles pertaining to different qualities, such as pore size, surface properties, and shape, in such a way that they would be utilized as protectants or for exact delivery through encapsulation, and adsorption of an active ingredient [8]. There is a possibility for nanotechnology in agriculture to create and give another age of fungicides and different active ingredients for fungal disease control in plants, as presented in Figure 1.

The application of nanoparticles for plants protection could be achieved using two types of mechanisms: (a) as individual nanoparticles giving protection in plants; or (b) nanoparticles as fungicides transporters of different active ingredients, for example, fertilizers, which could be applied by soaking/drenching or by spraying onto the foliar tissue, roots, or seeds. Nanoparticles serve as a carrier and may give numerous advantages such as (i) enhanced solubility of low water-soluble fungicides; (ii) enhanced shelf-life; (iii) improved site-specific uptake in the targeted microbe; and (iv) reduced toxicity [9]. Other advantage of the nanocarrier system is the increment for the efficacy of the stability and activity of the nanofungicides under different ecological factors, essentially reducing the number as well as the quantity of applications, which accordingly diminishes harmfulness and decreases their expenses or cost.

Nanotechnology has played an important role in creating a footprint to develop several forms of formulations effectively. Up to now, nanotechnology in the area of agriculture has not reached its milestone because of the insufficient application of nanoproducts at commercial levels. Till today, only a few studies have been carried out in the fields. Therefore, there is a need for researches to shift towards the direction of testing on different crops, target fungus, and to carry out both short and long periods of field trials in order to make progress, and advances in the area of agronanotechnology. This work discusses the properties and synthesis of nanoparticles, new advancements in plant pathogenic fungal disease control by the use of nanoparticles alone as protectants, and nanoparticles as nanocarriers for fungicides. Moreover, using other nanoproducts such as agronanofungicides, *Zataria multiflora*, and ginger essential oils nanoformulations to control plant pathogenic fungi, as well as the biosafety and limitations of the nanoparticles applications, have been addressed. In this review, we have focused on the information that is more recent (last 5 years) and also on papers before 2016 (last 5tears), where we feel the information is not as rich. The majority of the papers discuss the synthesis, advantages, and efficacy of nanomaterials to control plant pathogenic fungi. Few papers discuss the phytotoxicity and limitations of nanomaterials applications.

## 2. An Overview on Nanoparticles

### 2.1. Definition, Properties, Synthesis and Uses

Nanoparticles are materials with at least one or more dimensions at the scale of 1–100 nm [10]. This definition has taken several materials to be named nanoparticles into account. Nanoparticles that are natural likewise occur in numerous forms, for example, oceanic salt sprays and volcanic dust [11]. Moreover, numerous viroid and viral particles fall in this definition of a nanoparticle. Natural nanoparticles are having different sizes and are irregular. Because of their huge surface-area-to-volume ratio and little size, they could be highly reactive and may absorb, bind, and convey mixtures of compounds, for example, DNA, small molecular drugs, proteins, probes, and RNA [12,13]. Besides the large surface area of nanoparticles, they also vary in different properties when compared with their counterparts. For example, gold is inert and is clearly a bigger structure, but could be reactive and reddish in color at the scale of nano size. Similarly, ZnO and TiO are usually found to be colorless at a nano size. Nanoparticles were found to melt at low temperatures and have high reactive potentials than their counterparts [13,14]. 

Nanoparticles are synthesized by various strategies and methods such as laser ablation, pyrolysis, emulsion, encapsulation, dispersion-precipitation, etc. [15,16]. New cycles and stages are developed quickly to the point that any portrayal is probably going to be obsolete soon. A lot of works have been carried out on the synthesis and preparation of nanoparticles in vivo by both microorganisms and plants [17]. The nanoparticles are categorized into several groups such as organic nanoparticles, inorganic nanoparticles, carbon base nanoparticles, and ceramic nanoparticles. The inorganic nanoparticles are further categorized into metal oxide and metal nanoparticles [18]. Likewise, carbon base nanoparticles are also further categorized into carbon nanotubes, fullerene, carbon nanofiber, carbon black nanoparticles, and graphene. These nanoparticles could also be grouped in terms of their dimension, such as two-dimension nanoparticles, three-dimension nanoparticles, and one-dimension nanoparticles [18,19]. The nanoparticles can be prepared by utilizing different approaches, e.g., bottom-up approach and top-down approach. The effects of different nanomaterials used and methods of their synthesis are listed in Table 1.

### 2.2. Mechanism of Action of Nanoparticles

Nanoparticles were found to play a vital role in suppressing the development and activities of different fungi [28]. Singh et al. [29] reported that, out of 15 micronutrients, CuSo_4_ and Na_2_B_4_O_7_ have shown a significant outcome in managing rust disease of peas in the field. Silver PVP/NPs were researched for their antifungal effect against many yeasts and molds (*Candida glabrata*, *C. tropicalis*, *Aspergillus niger*, *C. albicans*, and *C. krusei*); it showed a promising antifungal action on the microbes [30]. A nanoparticle (Zinc oxide) was efficient in controlling the postharvest disease caused by some fungal species (*Botrytis cinerea* and *Penicillium expansum*).

**Figure 2:**
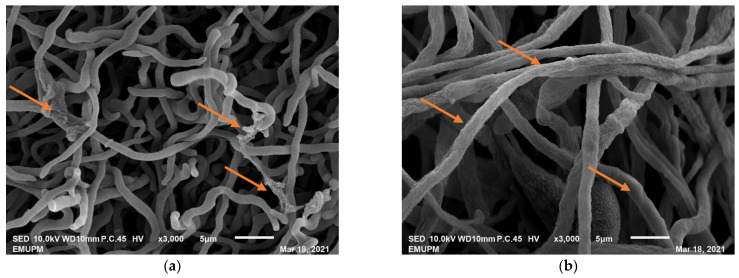
Micrographs of the scanning electron microscope (SEM) at a magnification of X3000. (**a**) Mycelia of *Pyricularia oryzae* fungus treated with chitosan nanoparticle, the mycelial growth was smaller with breakage at some points when compared to the control. (**b**) For the control, the mycelia of *Pyricularia oryzae* grown bigger, thicken, and without any breakage.

The treatment of nanoparticle, when applied, forestalled the development of conidiophores and conidia in *Penicillium expansum* fungus and caused distortion in the hyphae of *Botrytis cinerea* fungus. A nanoparticle (Zinc) was likewise found to be fungicidal; it has reduced the colonization of *Aspergillus flavus* at 15 mg mL^−1^ [28,31]. The fungicidal activity of nanoparticle (silver) was investigated by Krishnaraj et al. [32] and revealed that, at 15 mg L^−1^, it had fundamentally hindered the growth of various fungi that are pathogenic to different species of plants (*B. cinerea*, *Rhizoctonia solani*, *Curvularia lunata*, *Macrophomina phaseolina*, *Alternaria alternata*, and *Sclerotinia sclerotiorum*). Similarly, Malerba and Cerana [33] reported the potential mechanisms about the antifungal activity of chitosan, such as H^+^-ATPase activity inhibition, disruption of the plasma membrane, agglutination, inhibition of protein and messenger RNA, blockage of nutrient movement, and inhibition of toxin production. Moreover, based on our work on the use of chitosan nanoparticles to manage rice blast disease pathogen, our findings showed that chitosan nanoparticles at a concentration of 350 ppb have also shown strong antifungal activity against the *Pyricularia oryzae* fungus, as presented in Figure 2.

### 2.3. Types of Nanoparticles Used in Plant Pathogenic Fungal Control

#### 2.3.1. Nanoparticles as Protectants

Protectant nanoparticles are a material with a range of 10–100 nm; these nanoparticles have special structures and properties that are physically, biologically, and chemically unique [2,34]. Nanoparticles alone can be used on plant foliage, roots, or seeds for defense against different pathogens, such as fungi, insects, viruses, and bacteria. Nanoparticles that are metallic, such as copper, silver, titanium dioxide, and zinc oxide, have been widely investigated for their antifungal and antibacterial characteristics [33,35,36,37].

In recent years, silver nanoparticles have become popular because of their efficacy against bacteria and viruses [38]. The nanoparticles (silver) possess a strong antifungal effect against *Sclerotinia sclerotiorum*, *Alternaria alternata*, *Rhizoctonia solani*, *Macrophomina phaseolina*, *Curvularia lunata*, and *Botrytis cinerea* [32]. At the point when a silver nanoparticle was sprayed onto the leaves of the bean, total suppression of the sun-hemp rosette virus was noticed [39]. Elbeshehy et al. [40] stated that the best outcomes were recorded when the faba bean plant was inoculated with bean yellow mosaic virus, and sprayed 24 h after the infection with the silver nanoparticle. This was found to be more effective than the simultaneous application during inoculation or before disease symptoms appear (infection). Nanoparticles (silver) possess tremendous potential for fungal disease control against fungal microbes, yet there are critical obstacles related to them, such as their toxicity, soil interaction, and production [2].

Other widely utilized metallic nanoparticles are titanium dioxide, zinc oxide, gold, and copper [35]. Titanium dioxide and copper are widely being used as fertilizer with few investigations into disease control in plants, while a nanoparticle (Zinc oxide) was proven efficient in controlling the postharvest disease caused by some fungal species, such as *Botrytis cinerea* and *Penicillium expansum* [41]. The introduction of nanoparticles (polydispersed gold) by means of a mechanical abrasive has dissolved and melted the yellow mosaic virus particles and had given protection to the barley plant [42].

Chitosan is also a well-known nanoparticle with suitable biological characteristics, for example, biocompatibility, non-allergenicity, antimicrobial action, and biodegradability having low-toxic effects on humans and animals [43]. It also has the ability to actuate resistance to viruses in different tissues of plants by supporting them to resist several infections brought about by the mosaic virus of snuff, peanut, alfalfa, cucumber, and potato [44,45]. Nanoparticles of chitosan have possessed a significant antifungal characteristic, for example, controlling, tomato root rot, *Botrytis* bunch rot (grapes), *P. grisea* (rice plant), and *Fusarium* crown [46]; however, they are less effective against bacterial pathogens [33]. Antiviral activity has been observed on tobacco necrosis virus, tobacco mosaic virus, and bean mild mosaic virus [41]. Chitosan nanoparticles are promising as they appear to have a huge potential as nanocarriers [2].

#### 2.3.2. Nanoparticles as Nanocarriers

Nanoparticles are likewise regularly utilized as nanocarriers to encapsulate, entrap, and attach active particles to form a powerful formulation to be used in agroindustry. The regular nanoparticles which have been utilized as transporters or nanocarriers for fungicides are as follows: 

Silica nanoparticles could be prepared effectively to have a controlled shape, structure, and size, in order to ensure high delivery potentials [47]. They are normally prepared in a circular shape and having pore-like openings; e.g., mesoporous silica nanoparticles (MSNs) or porous hollow silica nanoparticles (PHSNs). MSN and PHSN usually load the fungicide in the internal center in order to prevent the active particles from escaping due to their volatile nature, and hence, give a precise delivery. Shell design of PHSNs ensures and prevents the active particles within the nanoparticles against UV light degradation. Different kinds of literature has reported that silicon has been utilized to improve plant resistance against different biotic and abiotic stresses and, along these lines, that nanoparticles of silica appear are a good choice for the production of different agro-industry products for fungal control [2]. Chitosan and solid lipid nanoparticles (SLNs) were used as the main carriers for disease control in plants.

Solid lipid nanoparticles (SLNs): These are like emulsions and are made out of solid lipids at ambient temperature. SLNs give a framework in order to entrap lipophilic active particles which do not require the utilization of solvents that are organic in nature [48]. Besides, SLNs can likewise give a controlled and effective release of different lipophilic molecules because of the diminished portability of these actives in the solid framework [49]. Surfactants are utilized to settle and cause the stability of the SLN when transferred into the water. Their principal disadvantages are that the actives could spill out of the structure in the period of storage and their low loading activity [50]. Tebuconazole (TBZ) ((RS)-1-(4-chlorophenyl)-4,4-dimethyl-3-(1H-1,2,4-triazol-1-ylmethyl) pentan-3-ol) and carbendazim (MBC) (methyl-2-benzimidazole carbamate) were commonly utilized in the agricultural industry for the management of different diseases caused by fungi [2]. Solid lipid nanoparticles serve as a carrier system that offers several potentialities including the reduced losses because of leaching, changes in the release profiles of bioactive compounds, and reduced toxicity in humans and the environment. These types of fungicide systems give an alternative for the management of fungal diseases in different plant species [2].

Chitosan nanoparticle: Chitosan is a biodegradable, biocompatible, and non-toxic compound [51], which possesses antifungal, antimicrobial, antioxidant, antiviral, bio-adhesion, adsorption enhancer, and anti-inflammatory properties [51]. The mode of action of chitosan against different microorganisms can be categorized into intracellular effects, extracellular effects, or both, depending on the targeting site of the antimicrobial effects [52,53,54,55]. Because high-MW chitosan is mainly unable to penetrate through the cell membrane and cell wall, its potential antimicrobial activities or effects include preventing nutrients from being absorbed from cells extracellularly, altering cell permeability, and acting as a chelator of essential metals [54,56]. For the low-MW chitosan, apart from having an extracellular antimicrobial effect, it also has the intracellular antimicrobial effect, thereby affecting protein synthesis, mitochondrial function, and RNA [54,57]. Chitosan was reported to have a negative impact on the microorganisms [58]. However, the antimicrobial activities of chitosan depend on its acetylation degree, microbial properties, and mass weight.

In the agricultural industry, chitosan nanoparticles alone are used as growth promoters and as strong antifungal agents to control different fungal infections. On the other hand, they are also used as nanocarriers for agrochemicals [59]. This nanocarrier system allows the encapsulation of active ingredients, either by covalent or ionic bonds or entangled in a polymeric matrix of chitosan, to form a potent nano delivery system of the formulation [51]. Chitosan with or without the consolidation of macronutrients can be used as a sustainable pesticide agent against pathogenic bacteria, viruses, and fungi. Chitosan with or without other active ingredients showed great potential as an option to utilize commercial fungicides against wilt and *Fusarium* head blight disease in chickpea and wheat plants, blast leaf in rice, post-flowering stalk rot in maize, leaf spot in maize, and blast disease in finger millet [59].

Chitosan possesses low dissolvability in water, because of its hydrophobic characteristics. Accordingly, chitosan is regularly combined with an organic, inorganic, and copolymer, in order to improve its dissolvability [60]. Chitosan has hydroxyl and reactive amine groups, permitting alteration, ionic interaction, and graft reactions, which improve the characteristics of chitosan. Chitosan clings well with the epidermis of plant stems and leaves, by enabling the take-up of the bioactive particles and prolonging the time of the contact [60].

##### Nanoparticle as a Carrier of Different Fungicides

The initial investigations on nanofungicides were carried out by fusing fungicides into solid wood [61]. From that point forward, different studies on fungicides having antifungal characteristics were carried out with a variety of nanoparticles (Table 2). Several types of essential oils, chitosan-dazomet-hexaconazole, chitosan-dazomet, and chitosan-hexaconazole, were excluded from the groups of fungicides, and nine fungicide resistance action committee (FRAC) groups were investigated. The most generally studied nanoparticle transporters or carriers were silica, chitosan, and polymer mixes. Different species of fungi were utilized to check the efficacy of these nanofungicides. 

Hatfaludi et al. [62] utilized nanosized non-denatured empty cell which covers the gram-negative microbe (bacteria) and bacterial ghosts to enhance low water solubility of tebuconazole and the ability to adhere to the surface of the leaf. *Pectobacterium cypripedii* was selected as a nanosized bacterial ghost, because of its capacity to adhere to the host (plants). Out of the tested plants (soya, cabbage, cotton, rice, corn, and barley), a labeled fluorescent ghost with fungicide loading clung best to the leaves of rice (55%), while adherence to the leaves of the soya was found to be the least (10%) during the glasshouse trial. All the six plant species were compared with either ghost-loaded (tebuconazole) or the two commercial treatments of tebuconazole (EW 250 and WP 25) in order to control different fungal species [62]. The plant species that have not been exposed to rainfall showed maximum protection to the products (commercial). In the case of plant species that were exposed to heavy rain 1 h after the application, and inoculated with the fungus, most of the groups did not respond well and the same was observed with the commercial product. Likewise, when washed 24 h after the application, tebuconazole (ghost-loaded) showed a similar result, or above the treatments (WP 25); however, EW 250-treated controls were the most effective. A low water-soluble fungicide (Pyraclostrobin) was loaded onto the lactide chitosan copolymer nanoparticle at several concentrations [63]. Five and three days after the application on the plant, it was discovered that the nanofungicide was the same or was less effective in inhibiting the growth of *C. gossypii* fungus when compared to the pyraclostrobin (commercial). Increased inhibition was noticed on the seventh day after the application when compared to the active alone [64]. Kaempferol fungicide was also loaded into lecithin/chitosan; this has shown inhibition of about 67% efficacy after 2 months of storage on the petri dish containing *F. oxysporum* [64].

Some studies reported that chlorothalonil and tebuconazole have been loaded onto different nanoparticles in order to increase the low solubility of these fungicides after they have been loaded onto the solid woods [65,66]. The investigations studied the mass loss as a result of the decay of southern yellow pine wood from *Gloeophyllum trabeum* for 2 months. The chlorothalonil (hydrophobic) encapsulated onto different nanoparticles have been observed to be ineffective because of the stability and size of the formulations, the increase in concentration, the decay of the wood was minimized [61]. Tebuconazole and chlorothalonil were encapsulated using the surfactant-free method, which had given rise to a more stable aqueous solution with smaller median particle diameters, and an increased uptake in the wood [61]. There was no serious decay (less than 5%) observed when using a minimum concentration as compared to the commercial fungicide in birchwood challenged with *Trametes versicolor* and southern pine challenged with *G. trabeum* [65]; this showed that the more stable and smaller surfactant-free nanoparticle was, it gave high resistance to the disease (fungal decay) [65]. Dazomet, hexaconazole and chitosan nanoencapsulation yielded a more stable aqueous solution and small median particle diameters, significantly increasing uptake into oil palm plant and helping with the management of basal stem rot (BSR) disease [59]. Similarly, the encapsulation of hexaconazole into chitosan and dazomet into chitosan have tremendously increased uptake and assisted in curtailing the effect of *G. boninense* in oil palm plants [59].

Essential oils (EOs) have fungicidal characteristics but were found to evaporate easily when applied in commercial use (large scale). Janatova et al. [67] have encapsulated five different EOs ingredients onto MSN and showed the significant antifungal effects two weeks after infection on *A. niger* pathogen. Likewise, SLNs were also applied to stabilize the essential oil of *Zataria multiflora* and had provided protection to six different species of fungi [68].

### 2.4. Leaching and Phytoxicity of Nanoparticles

Leaching is the movement of chemicals and water via soil and is considered as the main issue associated with pesticides; however, only a few investigations were carried out on this aspect. Wanyika [90] loaded metalaxyl fungicide into mesoporous silica nanoparticles (MSNs) and noticed that there was leaching in soil between an encapsulated metalaxyl (11.5% release) and free metalaxyl (76%) in one month. In water, the metalaxyl (encapsulated) showed an increased release rate (47%) when compared to (11.5%) that was observed within the soil, which thus justifies the advantage of testing it in the agro sector. Campos et al. [69] studied two kinds of nanoparticles, including polymeric, and investigated the cytotoxic effect of tebuconazole and carbendazim loaded into the nanoparticle. A decrease in the toxicity of fungicide loaded with nanoparticles was noticed in fibroblast and preosteoblast mouse cell lines. Some experiments on leaching in soil have shown that, when the nanoparticle was added, it decreased the release rate of the fungicide in soil layer when compared with the commercially formulated fungicides. Wang et al. [77] used difenoconazole and azoxystrobin loaded onto poly (lactic acid) and poly (butylene succinate) shells to treat zebrafish for 96 h; reduced toxicity as compared to the other forms of formulations was noticed. Nanosized calcium carbonate which carries validamycin was found to possess slow release of the actives [87]. The encapsulation of validamycin into nanoparticles has shown low effect than validamycin alone, for a period of 1 week against *R. solani* under in-vitro conditions. More so, 14 days after, the formulation of nanoparticles gave a better result than the active alone, which has proved the efficiency of the nanoformulations over a long time interval. Kumar et al. [74] reported significant inhibition of fungal mycelium where a carbendazim-loaded polymeric nanoparticle was applied against *A. parasiticus* and *F. oxysporum*, when compared to only carbendazim. The study on phytotoxicity reaffirms that the nanoformulation of carbendazim was found to be safer for root growth and germination of *Zea mays*, *Lycopersicum esculentum*, and *Cucumis sativa* plant seeds. Zhao et al. [75] utilized MSNs in loading of pyrimethanil, and investigated its uptake in cucumber for seven weeks. The dosage and uptake of fungicide-loaded MSN in the cucumber plant leaves had practically no impact on the dispersion and dissemination rate in the plants. Their investigation has reported that pyrimethanil-loaded MSN has a negligible danger of accumulating in the eatable portion of cucumber plants. Zhao et al. [75] has promoted our insight and comprehension of the dissemination and movement of MSN loaded with fungicides when sprayed on the leaves.

### 2.5. Limitations of Nanoparticles

The majority of the conventional approaches used to synthesize nanoparticles have some limitations, such as the generation of waste and the use of toxic chemicals, which are not friendly to the environment [98,99]. SLNs have some disadvantages because of their crystalline structure, they have low drug loading efficiency, and there is a possibility of drug expulsion as a result of the crystallization process at the storage conditions. Another limitation is the initial burst release which mainly occurs with the formulations [98]. In SLNs, the drug molecules orient between the glycerides or fatty acid chains, and during the periods of storage, could cause some polymorphic changes in solid lipid structures, and there is a chance of expulsion of the previously dissolved drug within the SLNs [98,99]. Presently, there are no human safety data available, and there could also be a change in drug release profile due to lipase degradation in some lipid matrix components. The surface characteristics, such as functional groups, the presence of some molecules, and charge, can affect the biocompatibility of MSNs [98,99,100]. Nanoparticles having a positive charge on the surface can provide significant cytotoxicity when compared to the anion and neutral species. MSNs having negative zeta potential could be associated with the serum opsonin [101]. Another issue is the number of -SiOH groups at the MSNs surface layer. This functional group could have a negative interaction with the biological molecules, such as lipids, plasma proteins, and cellular membranes, that destroy the structure of the biomolecules. As such, surface modification is the integral step in the modification of surface reactivity in order to improve biocompatibility and broaden the application of MSNs [98].

Despite the favorable chemical and physical characteristics of metal nanoparticles (MtNPs), the complex structure of soil and crop ecosystems indicated that the environmental behaviors of the nanoparticle are not yet fully predictable after application, and this is a serious issue [102,103]. Hence, before thoroughly using their potential, it is important to assess the impacts as well as the interaction within the living system. Therefore, the screening of nanoparticles is key in order to evaluate the potential toxicity and to fully understand the mechanism of action in order to prevent their negative impacts in the future [103]. The nanoscale dimensions of MtNPs, which determine the important properties, could also increase their potential negative impacts [102]. The MtNPs toxicity is affected by different factors, such as their binding specificity to biological sites and solubility [103]. Some researchers have shown the unpleasant aspect of long-term contact or exposure to some MtNPs, such as AgNPs and AuNPs. In a work by Vecchio et al. [102,103], the toxicity of AuNPs in *Drosophila melanogaster* (in vivo) was assessed. Because of the mutation that could be passed on to the offspring, a significant phenotypic change was noticed in the later generations of *Drosophila* after being treated with AuNPs; this had indicated the severity of AuNP toxicity [102]. These findings provided important evidence regarding the negative impacts of AuNPs on the development and growth of the organisms. These investigations have also unveiled the need for the assessment of the toxicological characteristics of nanoparticles and the need for nanoscience researchers to develop a biocompatible nanoparticle without any negative effects on the environment and human health [103].

## 3. Prospectives of Nanoformulations in Managing Plant Pathogenic Fungi

### 3.1. Agronanofungicides Formulations

The consolidation of agronanochemicals in the production of crops is fundamentally founded on the control of some plant diseases. Like nanosensors, the investigation in this area has quite recently been initiated [104]. The appraisals of disease were assessed based on the antifungal activity of agronanofungicides on *G. boninense* fungus (in vitro). Notwithstanding, not many investigations on the glasshouse or field assessment of utilizing agronanofungicides in controlling fungal diseases were carried out. A system of fungicide nanocarrier utilized by Mustafa et al. was designed by entrapping the fungicide in aluminum/zinc-layered double hydroxide (Al-Zn-LDH) by means of the ion exchange technique [104]. The type of fungicides utilized are dazomet and hexaconazole, which were recently demonstrated to be powerful on the fungus *G. boninense* [105]. Hexaconazole-controlled delivery characteristics were accomplished in H-Zn-Al-LDH at the EC_50_ of 30.0 ± 2.9 ng/mL. Then again, investigations on the phytotoxicity of D-Zn-Al-LDH and H-Zn-Al-LDH were carried out on oil palm plants; these results showed that both the two different types of formulations have the potential to bring down the phytotoxic impact when compared with their counterpart. In the interim, prior research conducted by [106] encapsulated a similar fungicide (dazomet and hexaconazole) onto chitosan nanoparticles in the development of a viable system of fungicide nanocarrier. The three types of formulation used include chitosan-hexaconazole-dazomet nanoparticles (CHDEN) [54,86], chitosan-dazomet nanoparticles (CDEN) [106], and chitosan-hexaconazole nanoparticles (CHEN) [107]. The study reported that the size of the particle can be modified by adjusting the concentration of sodium tripolyphosphate (TPP). Additionally, it was observed that the smaller the size of the particles, the higher the antifungal effect against the fungus (*G. boninense*) [108]. The least EC_50_ accomplished for CHDEN was 3.5 ± 1.0 ng/mL. Likewise, the lowest value of EC_50_ accomplished for CDEN and CHEN were 13.7 ± 1.8 and 4.6 ± 1.6 ng/mL, respectively. Similarly, Lee et al. stated that the antifungal activity of nanoemulsion (phenazine) on *G. boninense* with a 70.74% rate of inhibition was accomplished at 1000 μg/mL [109,110,111]. Shepros^®^ developed a new product, which was called *Ganoderma* eliminator on the basis of the nano-colloidal antifungal agent. Its key ingredients are Nano Alpha 10, nanosilver, derivatives of plants, and food additives. This product was found to infiltrate through inaccessible regions, for example, the septa of the fungi, and could repress the production of ergosterol in the cell membrane of the fungi [111].

### 3.2. Zataria multiflora Essential Oils Based Nanoformulations: For Controlling Fungi

The production of essential oils in plants is mainly for defense purposes against pathogenic microorganisms [112]. Essential oils have many benefits such as quick decomposition and with broad antifungal spectrum compared to conventional fungicides, low toxicity, and bioaccumulation. Nanoencapsulation is a nanocarrier system that is used for the encapsulation of bioactive substances [113]. It can improve the antifungal efficacy of bioactive compounds (essential oils) by the increase in cell interactions among the microorganisms and nanoparticles, because of the small size which improves the cellular uptake. Nanoencapsulation in solid lipid nanoparticles (SLNs) is an efficient technique that enhances the application of essential oils as an antifungal agent [113,114]. SLNs are novel drug delivery systems for cosmetic and pharmaceutical drug active ingredients [64]. SLNs have unique properties, such as a large surface area, high drug loading, and small size. Their sizes are in the range of 50–1000 nm. SLNs can improve the solubility of essential oil(EO) in water, protect the EO against environmental conditions such as light, oxygen, acidity, and moisture, improve the controlled release of the EO, and increase the bioavailability of entrapped bioactive [64]. 

*Zataria multiflora* essential oil-loaded solid lipid nanoparticles (ZE-SLNs) were proved to be efficient in managing several pathogens (fungi). The antifungal effect of ZE-SLNs and *Z. multiflora* essential oil (ZEO) was evaluated by many researchers (in vitro test) [68,113]. The findings showed that the ZE-SLNs and ZEO had 79 and 54% inhibition against the growth of some fungal species, respectively. The minimum inhibitory concentration (MIC) for in vitro test on the fungal pathogens (*A. niger*, *A. flavus*, *A. ochraceus*, *R. solani*, *R. stolonifera*, and *Alternaria solani*) showed that ZEO was less effective which found to inhibit the growth at 200, 300, 300, 200, 200, and 200 ppm, respectively, and that ZE-SLNs was more effective which found to inhibit the growth at 200, 200, 200, 50, 50 and 100 ppm, respectively. The antifungal efficacy of ZE-SLNs was significantly more than ZEO. Moreover, Moghimipour et al. [68] formulated EO of *Z. multiflora* by using SLNs based on different techniques and stated that *Z. multiflora* (Labiatae) found in Iran, Afghanistan, and Pakistan [106] has several potentialities against different bacteria and fungi [68].

The availability of phenolic compounds such as Carvacrol and Thymol are the major constituents of *Zataria multiflora* essential oil that inhibit the growth of *Aspergillus flavus* fungus. This essential oil nanoemulsion has a very strong anti-fungal activity with minimum inhibitory concentration (MIC) and minimum fungicidal concentration (MFC) of 100 ppm, respectively [113]. Based on these results, ZEO is an appropriate and potentially natural alternative for managing *A. flavus* [113]. In another study, the in vitro study had also shown a sustained and controlled release of *Z. multiflora* essential oils (ZEO) for 40 days. The strong activity of ZEO, after being encapsulated in chitosan nanoparticles (CSNPs) under both in vivo and in vitro conditions in comparison to the unmodified ZEO, was observed on the fungus *B. cinerea* [114]. The in vivo study had also revealed that the encapsulated *Zataria* essential oils at the concentration of 1500 ppm had shown a promising activity by decreasing both the disease incidence and disease severity of *Botrytis*-inoculated strawberries within the 7 days of storage at a temperature of 4 °C. This was then followed by two to three more days at a temperature of 20 °C. These findings have unveiled the important role of CSNPs that served as a controlled release system for *Zataria* EOs in order to enhance antifungal efficacies [114]. 

### 3.3. Ginger Essential Oils-Based Nanoformulations: For Controlling Fungi

**Figure 3:**
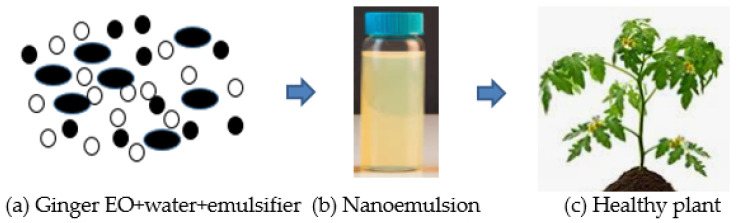
(**a**) Combination of ginger essential oil (EO), water, and emulsifier (tween-80). (**b**) The final product of ginger EO nanoemulsion after the formulation. (**c**) The application of ginger essential oil (EO) formulation is promising and could be utilized to protect the plant from fungal infections.

The delivery system of Eos, such as microemulsions, nanoemulsions, liposomes, and solid lipid nanoparticles, are designed for enclosing different compounds (natural bioactive) to improve antifungal efficacy [115,116]. Nanoemulsion is the dispersal of nanoparticles comprising of two different fluids that are insoluble, specifically water and oil, one of which is dispersed by a surfactant, as presented in Figure 3. Surfactant is needed in order to develop a formulation of nanoemulsions for interfacial layer rigidity, droplet quality under 100 nm, and droplet size reduction [117,118]. The utilization of EOs is very much designed to make explicit qualities implied for suitable uses [119,120] to manage the diseases of fungi. The decrease to the nanometric scale of the drop size could increase the zone of the substrate which then creates contact with the fungal pathogen to bring about cell death and lysis. The constituents of EOs can get to the pathways of the cell membrane due to their surface-to-volume proportions, physical characteristics, sizes, degrees of selectivity, and chemical stabilities, consequently setting the movement of EOs to arrive at their target areas [115].

The encapsulation innovations and controlled techniques for discharge have changed the utilization of nanotechnology-based ginger EOs as an antifungal and antibacterial for the conservation of different crops [121]. It is an effective delivery framework that may only be delivered when it is required, bringing about more prominent conservation of crops and lowering the costs of crop cultivation [122]. Some industries worldwide are aiming at formulating nanofungicides for conveyance by means of nanoencapsulation into the target tissue of plants. Numerous formulations are being developed to contain nanomaterials. The materials dissociate in water in order to improve their efficiencies [123]. Accordingly, they are utilized as nanoscale particles that comprise antifungal nanoparticle suspension that could be advantageously mixed with various media such as liquids, creams, and gels [123]. Previous investigations had uncovered that diverse nanoparticles could affect different pathogens. Thus, it is important to utilize nanoscale to develop new formulations from natural products, such as Eos, for fungicides [124]. Nanoemulsions that contain citral-EOs can disturb and enter the lipid structure of the cell wall (fungi). It brings about cell membrane annihilation and protein denaturation; this is followed by conformational changes, cell death, and cytoplasmic leakage. An effective system of delivery of useful particles from EOs would work in the treatment of fungal diseases of plants [124]. Mahdavi et al. [125] uncovered that polymeric nanofibers containing ginger EOs showed the consistent and nonstop delivery of the successful compound of EOs loaded onto the nanofibers which become a remarkable tool to control plant pathogens.

The main mechanism that mediates the cytotoxic impacts of ginger essential oils is the activation of cell death by promoting necrosis and apoptosis processes, loss of essential organelles, and the cell cycle suspension [126]. Many activities occur due to the low molecular weight of the major components made up of EOs that permit them to enter the cell membrane, lipophilic nature, modification of membrane composition which makes the membrane lower the ATP production, change in pH gradient, and rupturing of mitochondria which leads to the death of the cell [127]. Ginger EOs formulation could affect cytoplasm activity by disturbing the processes of respiration within the cells of the organism [128]. The efficacy of mitochondria can be impeded by reducing the functions of mitochondrial dehydrogenases associated with the biosynthesis of ATP, such as malate dehydrogenase, succinate dehydrogenase, and lactate dehydrogenase [129]. Abdullahi et al. [97] stated that the EOs from medicinal plants, such as ginger and oregano (*Origanum syriacum* var. bevanii (Holmes), were found to be effective in managing late blight disease of tomatoes. The treatment by using EOs has caused eased plasma membrane permeability and loss of cell wall integrity together with morphological changes in the mycelia of the targeted fungus. Ginger EOs is capable of acting as an antifungal agent which could inhibit the synthesis of ATP and disrupt the cycle in the mitochondria [97]. The mitochondria were altered due to the significant degradation of the internal content and a reduction in the cristae of the mitochondria.

## 4. Biosafety of Nanoparticles

This is a principal concern in the utilization of nanotechnology in the management of fungal diseases in plants [28]. A few uncertainties exist with respect to the long-term impact of utilizing nanofungicide formulations on human health and the environment [130,131,132,133]. Consequently, there is a need to assess the chance of inhaling the nanofungicide at the time of spray by the farm laborers. Shi et al. [134] studied the toxicity of chlorfenapyr (nanopesticide) on mice and expressed that the chlorfenapyr formulation at 4.84–19.36 mg kg^−1^ showed less toxicity than the conventional formulation on the mice. Hence, nanoformulation could lessen the impact on the environment and humans than the conventional fungicide [111,134].

Nanoformulations are seen to be safer and friendlier to the environment in disease control, yet a high level of NPs toxicity incidentally delivered to the environment could cause negative effects on other microbes and man [111]. The toxicological impacts of nanomaterials on soil microbes and plants have been generally studied. Notwithstanding, the nanotoxicity impacts of plant–soil systems of interaction are still not generally known [111]. There are numerous knowledge gaps on the agroecotoxicity of NPs; more so, there are numerous uncertain issues and new difficulties concerning the biological impacts. Mousa et al. [134] stated that there is a need to study the phytotoxic effect of seeds that are exposed to various concentrations of NPs; this involves the phytotoxicity investigation on germination, root length, and NPs uptake within the plant systems [96,135]. The application of nanosized silica-silver particles in the field helped in managing powdery mildew disease in cucurbits; about 100% disease management was obtained at 21 days after the application [136]. The NPs were discovered to be phytotoxic at a high concentration (3200 ppm) when applied in pansy and cucumber plants. Comparative investigation to convey the NPs to the target location of an infected plant was conducted by Corredor et al. [137]. The impact of NPs on various species of plants differs, and both the negative and positive impacts of this, have been discovered. The NPs may cause negative and positive impacts [138,139,140] on the root extension, which depends upon the species of plants (cucumber, soybean, corn, carrot, tomato, and cabbage). TiO_2_ and ZnO manufactured nanomaterials (MNMs) affected the microbe’s community, biomass, and their diversity in the soil. Together, such reports infer that the soybean that is exposed to MNMs may be directly affected or via interaction of plant and microorganism, which includes nitrogen-fixing symbioses association that is sensitive to some metals [141,142]. Again, the phytotoxicity investigations on D-Zn-Al-LDH and H-Zn-Al-LDH were carried out on the seedlings of oil palm, and the results showed that both had the potential to reduce the phytotoxic impact when compared with their conventional counterparts [111]. To understand the potential advantages of applying NT to agriculture, the initial step to determine the transport and penetration of NPs in plants is required [143]. Since nanomaterials are brought into the soil because of human activities, they can penetrate the soil through the biosolids amended soils and atmospheric routes. The transport and penetration of NPs in the entire plant were assessed by Gonz_alez-Melendi et al. [144]. The findings indicate the potential of NPs to deliver different substances that are inhibitory to the plant fungal pathogens. Many works are required to explain the interaction between plants, phylloplane microflora, nanomaterials, soil micro-organisms, and endophytes, as well as both pathogenic and beneficial effects on the health of plants. Moreover, further investigations are required in order to develop bioindicators that would not only evaluate the effect of NPs on the environment, but also recommend different designs as well as models for the evaluation [130]. 

## 5. Conclusions

Nanotechnology can increase potentialities for application in the area of agriculture and can change the current technique utilized in controlling plant pathogenic fungi. Nanofungicides development could give some potentialities such as enhanced efficacy and bioavailability of fungicides, reduced toxicity, and increased solubility of low water-soluble fungicides, and can also target delivery of the actives and precise release and enhance the shelf-life of the actives. Different types of nanoparticles and other forms of nanomaterials, such as agronanofungicides, *Z. multiflora* and ginger essential oils nanoformulations, were found to be effective and safe in the management of plant pathogenic fungi on a variety of crops. Due to the crystalline structure of SLNs which cause possible drug expulsion as a result of the crystallization process at the storage conditions, there is a need to enlighten the stockholders on the most suitable temperature for storing the SLNs; this will help in addressing the issue of drug expulsion. The nanoparticles (MSNs) having a positive charge on the surface can provide a significant cytotoxic effect when compared to the anion and neutral species. Therefore, this limitation could be overcome by educating the stockholders on which type of nanoparticle they are to buy or supply to their customers. The stockholders should always try to patronize the anion and neutral species of nanoparticles to overcome the issue of cytotoxicity. Some investigations have shown the negative aspect of long-term contact or exposure to some MtNPs, such as AgNPs and AuNPs. Therefore, this limitation could be addressed by enlightening the stockholders to be supplying the green or nanoparticles synthesized from natural products to their customers in order to make to environment safe and to prevent humans as well as other plants, microorganisms, and animals free from the risk of its toxicity. Biologists, as well as material researchers, need to work intently and bring in experts from different areas to obtain a more profound understanding of the major interaction and mechanisms in a system of bio-nanotechnology. It is likewise essential to choose a solid and reproducible framework to carry out efficacy and biocompatibility investigations at the organism, pest host ecosystem, and cellular levels.

## Figures and Tables

**Table 1:** Nanomaterials, methods of nanoparticles synthesis, effects and their uses.

Nanomaterial	Preparation Method	Advantages	Disadvantages	Effect	Source(s)
Organic					
Lipid Liposomes Lipopolyplexes Solid lipid nano-particles	Chemical: sonochemisty, reverse phase evaporation High-pressure homogenization	It involves the use of less toxic compounds, and the delivery of DNA, xenobiotics, pesticides, essential oils, and transfection	It requires substantial energy for effective disintegration of high-solid waste	Cytotoxicity	[20]
Carbon nanotubes, Nanofibers, Carbon nanospheres, activated carbon, nanodots, graphene oxide and graphene layer	Arc-discharge, laser ablation, pyrolysis, chemical vapor deposition, and Carbonization	Biocatalysts, sensing, neural/orthopedic implants atomic force microscope probes	It requires the use of low pressure and noble gasses	Antimicrobial effect	[21]
Synthetic Dendrimers (PAMAM, PPI) Polyethylene oxide Polyethylene glycol Polylactides Polyalklycyanoacrylates	-	Delivery of therapeutic/ diagnostic agents, pesticides delivery of DNA/RNA	Short half-lives, and lack of targeting capability	Cytotoxic effect	[22]
Polymeric Natural Cellulose, Starch Gelatin, Albumin Chitin, chitosan	Chemical: suspension, emulsion, dispersion -precipitation	Biocompatible, biodegradable non-toxic for drug delivery delivery of DNA/RNA	Emulsions are thermodynamically unstable and therefore must be formulated to stabilize the emulsion from the separation of the two phases	Non-toxic/non-cytotoxic	[23]
Inorganic					
Clay Montmorillonite layered double hydroxides	Physical: exfoliation co-precipitation	Delivery of pesticides, fertilizers, plant growth promoting factors	-	inhibiting and synergistic effects	[24]
Metal nanoparticles AgO, TiO_2_, ZnO, CeO_2_; Fe_2_O_3_ FePd, Fe–Ni (magnetic); Silica; CdTe, CdSe (QDs)	Physical: Arc-discharge, high energy ball milling, laser pyrolysis/ablation. Chemical: electrochemical, chemical vapor deposition sonochemistry, microemulsion sol-gel, reverse precipitation	Photothermal therapy, imaging studies, delivery of biomolecules (proteins, peptides nucleic acids), biosensors, diagnostic procedures, implants, pesticide degradation	It requires substantial energy for effective disintegration of high-solid waste, and the use of noble gas	Positive effect by promoting the growth of plants	[20]
Magnetic type					
Magnetic nanoparticle	Physical vapor deposition, mechanical attrition and chemical routes from solution	Photothermal therapy, Imaging studies, diagnostic procedures	special apparatus and formation of highly toxic gaseous as by-products	-	[18]
Biosynthesized type					
Biosynthesized nanoparticles (Silver and gold nanoparticles, Ag & Au NPs)	Ag^+^ ion reduction by culture supernatant of *E. coli*, gold ions reduction by Bacterial cell supernatant (Pseudomonas aeruginosa)	Delivery of pesticides and fertilizers.	Generally lower biosynthesis efficiency and lengthier production time Downstream processing of intracellular products is more complex and expensive	Antimicrobial effect	[25,26]
Nanocellulose and Cellulose nanocrystal	-	Degrading of biomass/bio-waste from oil palm	It has limited flexibility, low thermal stability, brittleness and low crystallization rate, which hinders its use	No cytotoxic and ecotoxic effects	[27]

PAMAM—polyamidoamine, PPI—polypropylene imine, QDs—semiconductor quantum dots.

**Table 2:** Summary of investigations using nanoparticles as actives targeting fungi and carriers of different fungicides.

Fungicide (FRAC Code)	Nanoparticles	Plant Species	Target Fungi	Soil Leaching or Toxicity	Advantages	Disadvantages	Source(s)
Carbendazim (1) Tebuconazole (3)	Polymeric and SLN	Bean seeds	-	Mouse fibroblast cells and soil sorption	A promising delivery system due to biocompatibility, and biodegradability of formulation constituents	Burst drug release from these nanocarriers may induce toxic effects	[69,70]
Chitosan–Dazomet- hexaconozole	Hexaconozole, Dazomet and chitosan	Oil palm	*Ganoderma boninense*	*-*	Control release of the actives	High concentration may cause phytotoxicity	[59]
Chitosan–Dazomet	Chitosan and Dazomet	Oil palm	*G. boninense*	*-*	It has high biocompatibility, and biodegradability of formulation constituents	High concentration may cause phytotoxicity	[59]
Chitosan–hexaconozole	Chitosan and hexaconozole	Oil palm	*G. boninense*	*-*	Effective nanodelivery system	High concentration may cause phytotoxicity	[51]
7 different volatile essential oils *	MSN	-	*A. niger*	-	Enhance the effectiveness of EOs against the fungal pthinkathogen	-	[67]
Kaempferol *	Lecithin/Chitosan	-	*F. oxysporum*	-	Improve bioavailability, time-dependent release, and therapeutic activity	The requirement of chemical cross-linking agents and/or repeated washing and precipitation steps.	[64,71]
*Zataria multiflora* essential oil *	SLN	-	*A. niger*, *A. ochraceus*, *A. flavus*, *R. solani* and *R. stolonifera* and *A. solani.*	-	A promising antifungal	-	[68]
Ferbam (M 03)	Gold	Tea leaves	-	-	Ease of transporting small molecules to the target pathogens	The challenges of using gold as nanodelivery system include biodistribution, pharmacokinetics and possible toxicity	[72]
Pyraclostrobin (11)	Chitosan/MSN	-	*P. asparagi*	-	A strong antifungal activity	High concentration may cause phytotoxicity	[73]
Carbendazim (1)	Chitosan/Pectin	Cucumber Maize Tomato	*A. parasiticus* and *F. oxysporum*	*-*	Control release	Phytotoxicity	[74]
Pyrimethanil (9)	MSN	Cucumber	-	-	It possesses a high surface area, large pore size, good biocompatibility and biodegradability	Cytotoxic effects	[75,76]
Carbendazim (1) Metalaxyl (4) Myclobutanil (3) Tebuconazole (3)	Magnetic nanocomposites	-	-	-	It possesses a distinctive active sites for various reactions	-	[77]
Prochloraz (3)	PHSN	Cucumber	B. cinerea	-	It facilitates the controlled nutrient transfer and increasing crop protection	High concentration may cause phytotoxicity	[78,79]
Clove essential oil *	Chitosan	-	*A. niger*	-	Reduce volatility and enhance fungal disease control	-	[80]
Tebuconazole (3) Propineb (M 03) Fludioxonil (12)	Silver	-	*B. maydis*	-	Control release of the actives	Low phytotoxic effect	[81]
*Cymbopogon martini* essential oil *	Chitosan	Maize grains	*F. graminearum*	-	Reduce volatility and enhance fungal disease control	-	[82]
Azoxystrobin (11) Difenoconazole (3)	PLA/PBS	-	-	Zebrafish	Improve biocompatibility	-	[83,84]
Pyraclostrobin (11)	MSN	-	*P. asparagi*	-	It possesses a high surface area, large pore size, good biocompatibility and biodegradability	Cytotoxic effects	[85]
Tebuconazole (3)	Bacterial ghosts	wheat, cucumber and Barley	*L. nodorum*, *P. teres*, *S. fuliginea* and *E. graminis*,	Barley (yellowing and necrosis)	It possesses a multifunctional delivery platforms	-	[62,86]
Validamycin (26)	PHSN	-	-	-	It facilitates the controlled nutrient transfer and increasing crop protection	High concentration may cause phytotoxicity	[87]
Validamycin (26)	Calcium carbonate	-	*R. solani*	-	Affordability and strong antimicrobial agent	Low toxicity	[87,88]
Tebuconazole (3)	PHSN	-	-	-	It increases crop protection	Phytotoxicity	[89]
Bioactive compounds from *Chaetomium* spp. *	PLA	-	-	-	Improve biocompatibility	-	[90]
Metalaxyl (4)	MSN	-	-	Soil sorption	It possesses a high surface area, large pore size, good biocompatibility and biodegradability	Cytotoxic effects	[90]
Pyraclostrobin (11)	Chitosan–PLA graft copolymer	-	*C. gossypii* Southw.	-	Improve biocompatibility	-	[63]
Flusilazole (3)	Chitosan–PLA graft copolymer	-	-	-	Improve biocompatibility	-	[91]
Bioactive compounds from *Chaetomium* spp. *	PLA	-	-	-	Enhance biodegradability	-	[90]
Tebuconazole (3) Chlorothalonil (M 05)	PVP and PVP copolymer	Southern pine sapwood	*G. trabeum*	-	It has excellent solubility in solvents of different polarities, good binding properties, and a stabilizing effect	The high absorption of humidity due to the strong hygroscopicity and hydrophilicity of the PVP can cause problems such as microbial contamination	[92,93]
Tebuconazole (3) Chlorothalonil (M 05)	PVP and PVP copolymer	Southern yellow pine	*G. trabeum*	-	It has excellent solubility in solvents of different polarities, good binding properties, and a stabilizing effect	The high absorption of humidity due to the strong hygroscopicity and hydrophilicity of the PVP can cause a problem such as microbial contamination	[66]
Tebuconazole (3) Chlorothalonil (M 05) KATHON 930 (32)	PVC	Southern and Birch yellow pine	*T. versicolor* (Turkey tail) *G. trabeum*	-	The highly biodegradable and water-soluble polymer	-	[61]
Tebuconazole (3) Chlorothalonil (M 05)	PVP and PVP copolymer	Southern and Birch yellow pine	*T. versicolor* (Turkey tail) *G. trabeum*	-	It has excellent solubility in solvents of different polarities, good binding properties, and a stabilizing effect	The high absorption of humidity due to the strong hygroscopicity and hydrophilicity of the PVP can cause problems such as microbial contamination	[65]
Eugenol oil	Eugenol oil Nanoemulsion	Seed cotton	*F. oxysporum* *F. vasinfectum*	-	Displayed better antifungal activity compared to its conventional form	-	[94]
Tea tree oil	Tea tree oil Nanocapsules	-	*Tricophyton rubrum*	-	Enhance the effectiveness of EOs against the fungal Pathogen	-	[95]
Chitosan	Chitosan nanoemulsion Chitosan	-	*Colletotrichum musae*, *C. gloeosporioides*	-	The control efficacy was efficient due to the slow and persistent release of the active components from the nanoparticles	It has phytotoxic effect, when high concentration is used	[96]
*Origanum dictamnus* Eos	Liposomes	-	*R. solani*, *S. sclerotiorum*, *C. lunata*	-	The control efficacy was about 80% due to the slow and persistent release of the active components from the nanoparticles	-	[97]
Chitosan	Chitosan	-	*Sclerotium rolfsii*, *Thanatephorus cucumeris*, *Fulvia fulva*, *R. stolonifer*	-	Enhance the effectiveness of chitosan against the fungal Pathogens	It has phytotoxic effect, when high concentration is used	[97]

In the above table, the investigated fungicides with the fungicides resistance action committee (FRAC) MoA group [2] and nanocarriers are also included. The investigation carried out on different plant species, against targeted fungi and exploring different environmental factors such as soil leaching and off-target toxicity are stated. * means natural fungicides without FRAC Code.

## Data Availability

All data is available in the main text.

## References

[1] Muhammad A., Isra N., Shahbaz T.S., Nasir A.R., Ehsan H., Muhammad U., Hamza S., Kiran F., Ehtsham A., Abdul Q. (2020). Nanoparticles: A safe way towards fungal diseases. Arch. Phytopathol. Plant Prot..

[2] Worrall E.A., Hamid A., Mody K.T., Mitter N., Pappu H.R. (2018). Nanotechnology for Plant Disease Management. Agronomy.

[3] Flood J. (2010). The importance of plant health to food security. Food Secur..

[4] Zaller J., Brühl C.A. (2019). Editorial: Non-target Effects of Pesticides on Organisms Inhabiting Agroecosystems. Front. Environ. Sci..

[5] Stephenson G.R. (2003). Pesticide Use and World Food Production: Risks and Benefits.

[6] Ghormade V., Deshpande M.V., Paknikar K.M. (2011). Perspectives for nano-biotechnology enabled protection and nutrition of plants. Biotechnol. Adv..

[7] Sinha K., Ghosh J., Sil P.C. (2017). 2—New pesticides: A cutting-edge view of contributions from nanotechnology for the development of sustainable agricultural pest control A2—Grumezescu, AlexandruMihai. New Pesticides and Soil Sensors.

[8] Maluin F.N., Hussein M.Z., Yusof N.A., Fakurazi S., Idris A.S., Hilmi N.H.Z., Daim L.D.J. (2020). Chitosan-Based Agronanofungicides as a Sustainable Alternative in the Basal Stem Rot Disease Management. J. Agric. Food Chem..

[9] Balaure P.C., Gudovan D., Gudovan I. (2017). Nanopesticides: A new paradigm in crop protection. New Pesticides and Soil Sensors.

[10] Sekhon B.S. (2014). Nanotechnology in agri-food production: An overview. Nanotechnol. Sci. Appl..

[11] Kadar E., Cunliffe M., Fisher A., Stolpe B., Lead J., Shi Z. (2014). Chemical interaction of atmospheric mineral dust-derived nanoparticles with natural seawater—EPS and sunlight-mediated changes. Sci. Total Environ..

[12] Waychunas G.A. (2009). Natural nanoparticle structure, properties and reactivity from X-ray studies. Powder Diffr. J..

[13] Albanese A., Tang P.S., Chan W.C. (2012). The effect of nanoparticle size, shape, and surface chemistry on biological systems. Annu. Rev. Biomed Eng..

[14] Wade E., Jason C.W. (2018). The Future of Nanotechnology in Plant Pathology. Annu. Rev. Phytopathol..

[15] Astruc D. (2008). Nanoparticles and Catalysis.

[16] Dreizin E.L. (2009). Metal-based reactive nanomaterials. Prog. Energy Combust. Sci..

[17] Costa Silva L.P., Oliveira J.P., Keijok W.J., Silva A.R., Aguiar A.R., Guimarães M.C.C., Braga F.R. (2017). Extracellular biosynthesis of silver nanoparticles using the cell-free filtrate of nematophagus fungus Duddingtonia flagans. Int. J. Nanomed..

[18] Ijaz I., Gilani E., Nazir A., Bukhari A. (2020). Detail review on chemical, physical and green synthesis, classification, characterizations and applications of nanoparticles. Green Chem. Lett. Rev..

[19] Abbasi E., Milani M., Aval S.F., Kouhi M., Akbarzadeh A., Nasrabadi H.T., Nikasa P., Joo S.W., Hanifehpour Y., Nejati-Koshki K. (2014). Silver nanoparticles: Synthesis methods, bio-applications and properties. Crit. Rev. Microbiol..

[20] Savun-Hekimoglu B.A. (2020). Review on Sonochemistry and Its Environmental Applications. Acoustics.

[21] Sari A.H., Khazali A., Parhizgar S.S. (2018). Synthesis and characterization of long-CNTs by electrical arc discharge in deionized water and NaCl solution. Int. Nano Lett..

[22] Filipczak N., Yalamarty S.S.K., Li X., Parveen F., Torchilin V. (2021). Developments in Treatment Methodologies Using Dendrimers for Infectious Diseases. Molecules.

[23] Mahato R., Narang A. (2018). Pharmaceutical Dosage Forms and Drug Delivery.

[24] Liu Y., Tong Z., Prud’homme R.K. (2008). Stabilized polymeric nanoparticles for controlled and efficient release of bifenthrin. Pest Manag. Sci..

[25] Simões M.F., Ottoni C.A., Antunes A. (2020). Biogenic Metal Nanoparticles: A New Approach to Detect Life on Mars?. Life.

[26] Vo T., Nguyen T., Huynh T., Vo T., Nguyen T., Nguyen D., Dang V., Dang C., Nguyen T. (2019). Biosynthesis of Silver and Gold Nanoparticles Using Aqueous Extract from *Crinum latifolium* Leaf and Their Applications Forward Antibacterial Effect and Wastewater Treatment. J. Nanomater..

[27] Islam M.N., Rahman F. (2019). Production and Modification of Nanofibrillated Cellulose Composites and Potential Applications. Green Composites for Automotive Applications.

[28] Mujeebur R.K., Tanveer F.R. (2014). Nanotechnology: Scope and Application in Plant Disease Management. Plant Pathol. J..

[29] Singh D., Kumar A., Singh A.K., Tripathi H.S. (2013). Induction of resistance in field pea against rust disease through various chemicals/micronutrients, and their impact on growth and yield. Plant Pathol. J..

[30] Bryaskova R., Pencheva D., Nikolov S., Kantardjiev T. (2011). Synthesis and comparative study on the antimicrobial activity of hybrid materials based on silver nanoparticles (AgNps) stabilized by polyvinylpyrrolidone (PVP). J. Chem. Biol..

[31] Jayaseelan C., Rahuman A.A., Kirthi A.V., Marimuthu S., Santhoshkumar T., Bagavan A., Gaurav K., Karthik L., Rao K. (2012). Novel microbial route to synthesize ZnO nanoparticles using Aeromonas hydrophila and their activity against pathogenic bacteria and fungi. Spectrochim. Acta Part A Mol. Biomol. Spectrosc..

[32] Krishnaraj C., Ramachandran R., Mohan K., Kalaichelvan P.T. (2012). Optimization for rapid synthesis of silver nanoparticles and its effect on phytopathogenic fungi. Spectrochim. Acta Part A Mol. Biomol. Spectrosc..

[33] Malerba M., Cerana R. (2016). Chitosan effects on plant systems. Int. J. Mol. Sci..

[34] Yang W., Peters J.I., Williams R.O. (2008). Inhaled nanoparticles—A current review. Int. J. Pharm..

[35] Kah M., Hofmann T. (2014). Nanopesticide research: Current trends and future priorities. Environ. Int..

[36] Gogos A., Knauer K., Bucheli T.D. (2012). Nanomaterials in plant protection and fertilization: Current state, foreseen applications, and research priorities. J. Agric. Food Chem..

[37] Mishra S., Singh H. (2015). Biosynthesized silver nanoparticles as a nanoweapon against phytopathogens: Exploring their scope and potential in agriculture. Appl. Microbiol. Biotechnol..

[38] Rafique M., Sadaf I., Rafique M.S., Tahir M.B. (2017). A review on green synthesis of silver nanoparticles and their applications. Artif. Cells Nanomed. Biotechnol..

[39] Jain D., Kothari S. (2014). Green synthesis of silver nanoparticles and their application in plant virus inhibition. J. Mycol. Plant Pathol..

[40] Elbeshehy E.K.F., Elazzazy A.M., Aggelis G. (2015). Silver nanoparticles synthesis mediated by new isolates of Bacillus spp., nanoparticle characterization and their activity against Bean Yellow Mosaic Virus and human pathogens. Front. Microbiol..

[41] Sadeghi R., Rodriguez R.J., Yao Y., Kokini J.L. (2017). Advances in nanotechnology as they pertain to food and agriculture: Benefits and risks. Annu. Rev. Food Sci. Technol..

[42] Alkubaisi N.A.O., Aref N.M.M.A., Hendi A.A. (2015). Method of Inhibiting Plant Virus Using Gold Nanoparticles. U.S. Patents.

[43] Cota-Arriola O., Onofre Cortez-Rocha M., Burgos-Hernández A., Marina Ezquerra-Brauer J., Plascencia-Jatomea M. (2013). Controlled release matrices and micro/nanoparticles of chitosan with antimicrobial potential: Development of new strategies for microbial control in agriculture. J. Sci. Food Agric..

[44] Kochkina Z., Pospeshny G., Chirkov S. (1994). Inhibition by chitosan of productive infection of T-series bacteriophages in the Escherichia coli culture. Mikrobiologiia.

[45] Pospieszny H., Chirkov S., Atabekov J. (1991). Induction of antiviral resistance in plants by chitosan. Plant Sci..

[46] Kashyap P.L., Xiang X., Heiden P. (2015). Chitosan nanoparticle based delivery systems for sustainable agriculture. Int. J. Biol. Macromol..

[47] Mody V.V., Cox A., Shah S., Singh A., Bevins W., Parihar H. (2014). Magnetic nanoparticle drug delivery systems for targeting tumor. Appl. Nanosci..

[48] Ekambaram P., Sathali A.A.H., Priyanka K. (2012). Solid lipid nanoparticles: A review. Sci. Rev. Chem. Commun..

[49] Borel T., Sabliov C. (2014). Nanodelivery of bioactive components for food applications: Types of delivery systems, properties, and their effect on ADME profiles and toxicity of nanoparticles. Annu. Rev. Food Sci. Technol..

[50] Tamjidi F., Shahedi M., Varshosaz J., Nasirpour A. (2013). Nanostructured lipid carriers (NLC): A potential delivery system for bioactive food molecules. Innov. Food Sci. Emerg. Technol..

[51] Maluin F.N., Hussein M.Z. (2020). Chitosan-Based Agronanochemicals as a sustainable alternative in Crop Protection. Molecules.

[52] Ke C.-L., Deng F.-S., Chuang C.-Y., Lin C.-H. (2021). Antimicrobial Actions and Applications of Chitosan. Polymers.

[53] Varlamov V.P., Mysyakina I.S. (2018). Chitosan in biology, microbiology, medicine, and agriculture. Microbiology.

[54] Kravanja G., Primozic M., Knez Z., Leitgeb M. (2019). Chitosan-based (Nano) materials for novel biomedical applications. Molecules.

[55] Hosseinnejad M., Jafari S.M. (2016). Evaluation of different factors affecting antimicrobial properties of chitosan. Int. J. Biol. Macromol..

[56] Ke C.L., Liao Y.T., Lin C.H. (2021). MSS2 maintains mitochondrial function and is required for chitosan resistance, invasive growth, biofilm formation and virulence in Candida albicans. Virulence.

[57] Seankamsorn A., Cherdthong A., So S., Wanapat M. (2021). Influence of chitosan sources on intake, digestibility, rumen fermentation, and milk production in tropical lactating dairy cows. Trop. Anim. Health Prod..

[58] Seankamsorn A., Cherdthong A., Wanapat M. (2020). Combining crude glycerin with chitosan can manipulate in vitro ruminal efficiency and inhibit methane synthesis. Animals.

[59] Maluin F.N., Mohd Z.H., Nor Azah Y., Sharida F., Idris A.S., Nur Hailini Z.H., Leona D.J.D. (2019). Enhanced fungicidal efficacy on Ganoderma boninense by simultaneous co-delivery of hexaconazole and dazomet from their chitosan nanoparticles. RSC Adv..

[60] Li M., Huang Q., Wu Y. (2011). A novel chitosan-poly (lactide) copolymer and its submicron particles as imidacloprid carriers. Pest Manag. Sci..

[61] Liu Y., Laks P., Heiden P. (2002). Controlled release of biocides in solid wood. II. Efficacy against Trametes versicolor and Gloeophyllum trabeum wood decay fungi. J. Appl. Polym. Sci..

[62] Hatfaludi T., Liska M., Zellinger D., Ousman J.P., Szostak M., Jalava K., Lubitz W. (2004). Bacterial ghost technology for pesticide delivery. J. Agric. Food Chem..

[63] Xu L., Cao L.-D., Li F.-M., Wang X.-J., Huang Q.-L. (2014). Utilization of chitosan-lactide copolymer nanoparticles as controlled release pesticide carrier for pyraclostrobin against Colletotrichum gossypii Southw. J. Dispers. Sci. Technol..

[64] Ilk S., Saglam N., Özgen M. (2017). Kaempferol loaded lecithin/chitosan nanoparticles: Preparation, characterization, and their potential applications as a sustainable antifungal agent. Artif. Cells Nanomed. Biotechnol..

[65] Liu Y., Laks P., Heiden P. (2002). Controlled release of biocides in solid wood. III. Preparation and characterization of surfactant-free nanoparticles. J. Appl. Polym. Sci..

[66] Liu Y., Laks P., Heiden P. (2002). Controlled release of biocides in solid wood. I. Efficacy against brown rot wood decay fungus (Gloeophyllum trabeum). J. Appl. Polym. Sci..

[67] Janatova A., Bernardos A., Smid J., Frankova A., Lhotka M., Kourimská L., Pulkrabek J., Kloucek P. (2015). Long-term antifungal activity of volatile essential oil components released from mesoporous silica materials. Ind. Crops Prod..

[68] Nasseri M., Golmohammadzadeh S., Arouiee H., Jaafari M.R., Neamati H. (2016). Antifungal activity of Zataria multiflora essential oil-loaded solid lipid nanoparticles in-vitro condition. Iran. J. Basic Med. Sci..

[69] Campos E.V.R., De Oliveira J.L., Da Silva C.M.G., Pascoli M., Pasquoto T., Lima R., Abhilash P., Fraceto L.F. (2015). Polymeric and solid lipid nanoparticles for sustained release of carbendazim and tebuconazole in agricultural applications. Sci. Rep..

[70] Ghasemiyeh P., Mohammadi-Samani S. (2018). Solid lipid nanoparticles and nanostructured lipid carriers as novel drug delivery systems: Applications, advantages and disadvantages. Res. Pharm. Sci..

[71] Liu L., Zhou C., Xia X., Liu Y. (2016). Self-assembled lecithin/chitosan nanoparticles for oral insulin delivery: Preparation and functional evaluation. Int. J. Nanomed..

[72] Hou R., Zhang Z., Pang S., Yang T., Clark J.M., He L. (2016). Alteration of the nonsystemic behavior of the pesticide ferbam on tea leaves by engineered gold nanoparticles. Environ. Sci. Technol..

[73] Cao L., Zhang H., Cao C., Zhang J., Li F., Huang Q. (2016). Quaternized chitosan-capped mesoporous silica nanoparticles as nanocarriers for controlled pesticide release. Nanomaterials.

[74] Kumar S., Kumar D., Dilbaghi N. (2017). Preparation, characterization, and bio-efficacy evaluation of controlled release carbendazim-loaded polymeric nanoparticles. Environ. Sci. Pollut. Res..

[75] Zhao P., Cao L., Ma D., Zhou Z., Huang Q., Pan C. (2017). Synthesis of Pyrimethanil-Loaded Mesoporous Silica Nanoparticles and Its Distribution and Dissipation in Cucumber Plants. Molecules.

[76] Jafari S., Derakhshankhah H., Alaei L., Fattahi A., Varnamkhasti B.S., Saboury A.A. (2019). Mesoporous silica nanoparticles for therapeutic/diagnostic applications. Biomed. Pharmacother..

[77] Wang X., Ma X., Huang P., Wang J., Du T., Du X., Lu X. (2018). Magnetic Cu-MOFs embedded within graphene oxide nanocomposites for enhanced preconcentration of benzenoid-containing insecticides. Talanta.

[78] Zhao P., Cao L., Ma D., Zhou Z., Huang Q., Pan C. (2018). Translocation, distribution and degradation of prochloraz-loaded mesoporous silica nanoparticles in cucumber plants. Nanoscale.

[79] Khoo K.S., Chia W.Y., Tang D.Y.Y., Show P.L., Chew K.W., Chen W. (2020). Nanomaterials Utilization in Biomass for Biofuel and Bioenergy Production. Energies.

[80] Hasheminejad N., Khodaiyan F., Safari M. (2019). Improving the antifungal activity of clove essential oil encapsulated by chitosan nanoparticles. Food Chem..

[81] Huang W., Wang C., Duan H., Bi Y., Wu D., Du J., Yu H. (2018). Synergistic Antifungal Effect of Biosynthesized Silver Nanoparticles Combined with Fungicides. Int. J. Agric. Biol..

[82] Kalagatur N.K., Ghosh O.S.N., Sundararaj N., Mudili V. (2018). Antifungal activity of chitosan nanoparticles encapsulated with Cymbopogon martinii essential oil on plant pathogenic fungi Fusarium graminearum. Front. Pharmacol..

[83] Wang Y., Li C., Wang Y., Zhang Y., Li X. (2018). Compound pesticide controlled release system based on the mixture of poly(butylene succinate) and PLA. J. Microencapsul..

[84] Casalini T., Rossi F., Castrovinci A., Perale G.A. (2019). Perspective on Polylactic Acid-Based Polymers Use for Nanoparticles Synthesis and Applications. Front. Bioeng. Biotechnol..

[85] Cao L., Zhang H., Zhou Z., Xu C., Shan Y., Lin Y., Huang Q. (2018). Fluorophore-free luminescent double-shelled hollow mesoporous silica nanoparticles as pesticide delivery vehicles. Nanoscale.

[86] Li R., Liu Q. (2020). Engineered Bacterial Outer Membrane Vesicles as Multifunctional Delivery Platforms. Front. Mater..

[87] Qian K., Shi T., Tang T., Zhang S., Liu X., Cao Y. (2011). Preparation and characterization of nano-sized calcium carbonate as controlled release pesticide carrier for validamycin against Rhizoctonia solani. Microchim. Acta.

[88] Mydin R.S.M.N., Zahidi I.N.M., Ishak N.N., Nik Ghazali N.S.S., Moshawih S., Siddiquee S. (2018). Potential of Calcium Carbonate Nanoparticles for Therapeutic Applications. Malays. J. Med. Health Sci..

[89] Qian K., Shi T., He S., Luo L., Cao Y. (2013). Release kinetics of tebuconazole from porous hollow silica nanospheres prepared by miniemulsion method. Microporous Mesoporous Mater..

[90] Wanyika H. (2013). Sustained release of fungicide metalaxyl by mesoporous silica nanospheres. J. Nanopart. Res..

[91] Dar J., Soytong K. (2014). Construction and characterization of copolymer nanomaterials loaded with bioactive compounds from Chaetomium species. J. Agric. Technol..

[92] Liu Y., Yan L., Heiden P., Laks P. (2001). Use of nanoparticles for controlled release of biocides in solid wood. J. Appl. Polym. Sci..

[93] Franco P., De Marco I. (2020). The Use of Poly (N-vinyl pyrrolidone) in the Delivery of Drugs: A Review. Polymers.

[94] Shao Y., Wu C., Wu T., Li Y., Chen S., Yuan C., Hu Y. (2018). Eugenol-chitosan nanoemulsions by ultrasound-mediated emulsification: Formulation, characterization and antimicrobial activity. Carbohydr. Polym..

[95] Flores F.C., De Lima J.A., Ribeiro R.F., Alves S.H., Rolim C.M.B., Beck R.C.R., Da Silva C.B. (2013). Antifungal Activity of Nanocapsule Suspensions Containing Tea Tree Oil on the Growth of Trichophyton rubrum. Mycopathologia.

[96] Kaushal M., Abd-Elsalam K.A., Prasad R. (2018). Role of microbes in plant protection using intersection of nanotechnology and biology. Nanobiotechnology Applications in Plant Protection.

[97] Abdullahi A., Ahmad K., Ismail I.S., Asib N., Ahmed O.H., Abubakar A.I., Siddiqui Y., Ismail M.R. (2020). Potential of Using Ginger Essential Oils-Based Nanotechnology to Control Tropical Plant Diseases. Plant Pathol. J..

[98] Casagrande M.G., De Lima R. (2019). Synthesis of Silver Nanoparticles Mediated by Fungi: A Review. Front. Bioeng. Biotechnol..

[99] Ahmed S., Ahmad M., Swami B.L., Ikram S. (2016). A review on plants extract mediated synthesis of silver nanoparticles for antimicrobial applications: A green expertise. J. Adv. Res..

[100] Makwana V., Jain R., Patel K., Nivsarkar M., Joshi A. (2015). Solid lipid nanoparticles (SLN) of Efavirenz as lymph targeting drug delivery system: Elucidation of mechanism of uptake using chylomicron flow blocking approach. Int. J. Pharm..

[101] Bahrulolum H., Nooraei S., Javanshir N., Tarrahimofrad H., Mirbagheri V.S., Easton A.J., Ahmadian G. (2021). Green synthesis of metal nanoparticles using microorganisms and their application in the agrifood sector. J. Nanobiotechnol..

[102] Cas tillo-Henriquez L., Alfaro-Aguilar K., Ugalde-Alvarez J., Vega-Fernandez L., Montes de Oca-Vasquez G., Vega-Baudrit J.R. (2020). Green synthesis of gold and silver nanoparticles from plant extracts and their possible applications as antimicrobial agents in the agricultural area. Nanomaterials.

[103] Chen H. (2018). Metal based nanoparticles in agricultural system: Behavior, transport, and interaction with plants. Chem. Speciat. Bioavailab..

[104] Mustafa I.F., Hussein M.Z., Saifullah B., Idris A.S., Hilmi N.H.Z., Fakurazi S. (2018). Synthesis of (hexaconazole-zinc/aluminum-layered double hydroxide nanocomposite) fungicide nanodelivery system for controlling *Ganoderma* disease in oil palm. J. Agric. Food Chem..

[105] Mustafa I.F., Hussein M.Z., Seman I.A., Hilmi N.H.Z., Fakurazi S. (2018). Synthesis of dazomet-zinc/aluminumlayered double hydroxide nanocomposite and its phytotoxicity effect on oil palm seed growth. ACS Sustain. Chem. Eng..

[106] Ariffin D., Idris S. Investigation on the control of *Ganoderma* with dazomet. Proceedings of the PORIM International Palm Oil Conference. Progress, Prospects Challenges Towards the 21st Century. (Agriculture).

[107] Maluin F.N., Hussein M.Z., Yusof N.A., Fakurazi S., Idris A.S., Hilmi Z., Hailini N., Jeffery Daim L.D. (2019). Preparation of chitosan–hexaconazole nanoparticles as fungicide nanodelivery system for combating *Ganoderma* disease in oil palm. Molecules.

[108] Maluin F.N., Hussein M.Z., Yusof N.A., Fakurazi S., Idris A.S., Hilmi N.H.Z., Jeffery Daim L.D. (2019). A potent antifungal agent for basal stem rot disease treatment in oil palms based on chitosan-dazomet nanoparticles. Int. J. Mol. Sci..

[109] Azhari M.A., Putri I.W., Pratama A.I., Hidayah R.E., Ambarsari L. (2019). Development of *Trichodermin* nanoemulsion based on medium chain triglycerides as antifungal of *Ganoderma boninense* in vitro. Curr. Biochem..

[110] Lee K.W., bin Omar D., bt Abdan K., Wong M.Y. (2016). physiochemical characterization of nanoemulsion formulation of phenazine and their antifungal efficacy against *Ganoderma boninense* PER71 in vitro. Res. J. Pharm. Biol. Chem. Sci..

[111] Maluin F.N., Hussein M.Z., Idris A.S. (2020). An Overview of the Oil Palm Industry: Challenges and Some Emerging Opportunities for Nanotechnology Development. Agronomy.

[112] Pinilla C.M.B., Lopes N.A., Brandelli A. (2021). Lipid-Based Nanostructures for the Delivery of Natural Antimicrobials. Molecules.

[113] Mohammadi A., Hashemi M., Hosseini S.M. (2015). Nanoencapsulation of Zataria multiflora essential oil preparation and characterization with enhanced antifungal activity for controlling Botrytis cinerea, the causal agent of gray mould disease. Innov. Food Sci. Emerg. Technol..

[114] Rahimi V., Hekmatimoghaddam S., Jebali A., Sadrabad E.K., Heydari A., Mohajeri F.A. (2019). Chemical Composition and Antifungal Activity of Essential Oil of Zataria Multiflora. J. Nutr. Food Secur..

[115] Adamu A., Ahmad K., Siddiqui Y., Ismail I.S., Asib N., Bashir Kutawa A., Adzmi F., Ismail M.R., Berahim Z. (2021). Ginger Essential Oils-Loaded Nanoemulsions: Potential Strategy to Manage Bacterial Leaf Blight Disease and Enhanced Rice Yield. Molecules.

[116] Prasad R., Kumar V., Prasad K.S. (2014). Nanotechnology in sustainable agriculture: Present concerns and future aspects. Afr. J. Biotechnol..

[117] Acharya A., Pal P.K. (2020). Agriculture nanotechnology: Translating research outcome to field applications by luencing environmental sustainability. NanoImpact.

[118] Echeverria J., de Albuquerque R.D.D.G. (2019). Nanoemulsions of essential oils: New tool for control of vector-borne diseases and in vitro effects on some parasitic agents. Medicines.

[119] Pedro A.S., Santo I.E., Silva C.V., Detoni C., Albuquerque E., Médnez-Vilas A. (2013). The use of nanotechnology as an approach for essential oil-based formulations with antimicrobial activity. Microbial Pathogens and Strategies for Combating Them: Science, Technology and Education.

[120] Lim C.J., Basri M., Omar D., Abdul Rahman M.B., Salleh A.B., Raja Abdul Rahman R.N.Z. (2012). Physicochemical characterization and formation of glyphosate-laden nanoemulsion for herbicide formulation. Ind. Crops Prod..

[121] Khot L.R., Sankaran S., Maja J.M., Ehsani R., Schuster E.W. (2012). Applications of nanomaterials in agricultural production and crop protection: A review. Crop Prot..

[122] Nirmala M.J., Nagarajan R. (2017). Recent research trends in fabrication and applications of plant essential oil based nanoemulsions. J. Nanomed. Nanotechnol..

[123] Rai M., Ingle A. (2012). Role of nanotechnology in agriculture with special reference to management of insect pests. Appl. Microbiol. Biotechnol..

[124] Lu W.C., Huang D.W., Wang C.C.R., Yeh C.H., Tsai J.C., Huang Y.T., Li P.H. (2018). Preparation, characterization, and antimicrobial activity of nanoemulsions incorporating citral essential oil. J. Food Drug Anal..

[125] Mahdavi V., Rafiee-Dastjerdi H., Asadi A., Razmjou J., Fathi Achachlouei B. (2018). Synthesis of *Zingiber officinale* essential oil-loaded nanofiber and its evaluation on the potato tuber moth, *Phthorimaea operculella* (Lepidoptera: Gelechiidae). J. Crop Prot..

[126] Sharifi-Rad J., Sureda A., Tenore G.C., Daglia M., Sharifi-Rad M., Valussi M., Tundis R., Sharifi-Rad M., Loizzo M.R., Ademiluyi A. (2017). Biological activities of essential oils: From plant chemoecology to traditional healing systems. Molecules.

[127] Dhifi W., Bellili S., Jazi S., Bahloul N., Mnif W. (2016). Essential oils’ chemical characterization and investigation of some biological activities: A critical review. Medicines.

[128] Akhtar M.S., Swamy M.K., Sinniah U.R. (2019). Natural Bio-Active Compounds.

[129] Nazzaro F., Fratianni F., Coppola R., De Feo V. (2017). Essential oils and antifungal activity. Pharmaceuticals.

[130] Mustafa M.A., Ali A., Manickam S., Siddiqui Y. (2014). Ultrasound-assisted chitosan–surfactant nanostructure assemblies: Towards maintaining postharvest quality of tomatoes. Food Bioprocess Technol..

[131] Zahid N., Maqbool M., Ali A., Siddiqui Y., Abbas Bhatti Q. (2019). Inhibition in production of cellulolytic and pectinolytic enzymes of Colletotrichum gloeosporioides isolated from dragon fruit plants in response to submicron chitosan dispersions. Sci. Hortic..

[132] Ali A., Cheong C.K., Zahid N. (2014). Composite Effect of Propolis and Gum Arabic to Control Postharvest Anthracnose and Maintain Quality of Papaya during Storage. Int. J. Agric. Biol..

[133] Ali A., Zahid N., Manickam S., Siddiqui Y., Alderson P.G. (2014). Double Layer Coatings: A new technique for maintaining physico-chemical characteristics and antioxidant properties of dragon fruit during storage. Food Bioprocess Technol..

[134] Mousa A.A., Hassan A., Mahindra R., Ernest S., Kamel A.A. (2015). Myconanoparticles: Synthesis and their role in phytopathogens management. Biotechnol. Biotechnol. Equip..

[135] Kumari M., Ernest V., Mukherjee A., Chandrasekaran N. (2012). In vivo nanotoxicity assays in plant models. Methods Mol. Biol..

[136] Park H.-J., Kim S.H., Kim H.J., Choi S.-H. (2006). A new composition of nanosized silica-silver for control of various plant diseases plant. Pathol. J..

[137] Corredor E., Testillano P.S., Coronado M.-J., González-Melendi P., Fernández-Pacheco R., Marquina C., Ibarra M.R., De La Fuente J.M., Rubiales D., de Luque A.P. (2009). Nanoparticle penetration and transport in living pumpkin plants: In situ subcellular identification. BMC Plant Biol..

[138] Khodakovskaya M., Dervishi E., Mahmood M., Xu Y., Li Z., Watanabe F., Biris A.S. (2009). Carbon nanotubes are able to penetrate plant seed coat and dramatically affect seed germination and plant growth. ACS Nano.

[139] Lin D., Xing B. (2007). Phytotoxicity of nanoparticles: Inhibition of seed germination and root growth. Environ. Pollut..

[140] Ge Y., Schimel J.P., Holden P.A. (2011). Evidence for negative effects of TiO_2_ and ZnO nanoparticles on soil bacterial communities. Environ. Sci. Technol..

[141] Lee S., Kim S., Kim S., Lee I. (2012). Effects of soil-plant interactive system on response to exposure to ZnO nanoparticles. J. Microbiol. Biotechnol..

[142] Degrassi G., Bertani I., Devescovi G., Fabrizi A., Gatti A., Venturi V. (2012). Response of plant-bacteria interaction models to nanoparticles. EQA.

[143] Nowack B., Ranville J.F., Diamond S., Gallego-Urrea J.A., Metcalfe C., Rose J., Horne N., Koelmans A.A., Klaine S.J. (2012). Potential scenarios for nanomaterial release and subsequent alteration in the environment. Environ. Toxicol. Chem..

[144] González-Melendi P., Fernández-Pacheco R., Coronado M.J., Corredor E., Testillano P.S., Risueño M.C., Marquina C., Ibarra M.R., Rubiales D., de Luque A.P. (2008). Nanoparticles as smart treatment-delivery systems in plants; assessment of different techniques of microscopy for their visualization in plant tissues. Ann. Bot..

